# Social Support and Peer Group Integration of Adolescents with Diabetes

**DOI:** 10.3390/ijerph18042064

**Published:** 2021-02-20

**Authors:** María de los Ángeles Núñez-Baila, Anjhara Gómez-Aragón, José Rafael González-López

**Affiliations:** Department of Nursing, Faculty of Nursing, Physiotherapy and Podiatry, Universidad de Sevilla, C/Avenzoar nº6, 41009 Seville, Spain; marnunbai@alum.us.es (M.d.l.Á.N.-B.); joserafael@us.es (J.R.G.-L.)

**Keywords:** type 1 diabetes mellitus, adolescents, social support, peer influence, role

## Abstract

The aim of this study was to examine, through the roles of peers with regards to diabetes, the relationship between the support perceived by adolescents with diabetes and their peer-group affiliation. This is a descriptive, phenomenological and retrospective study based on a qualitative methodology. In-depth interviews with 15 people aged 18–35 with type 1 diabetes mellitus diagnosed in their childhood or adolescence were carried out. Data was analyzed through the interpretation of general discourses. Peers have considerable influence on adolescents and provide them social support from different roles. The protective role basically offers emotional support and sends reminders of different aspects of the treatment, while the indifferent role does not meddle in any aspect related to the diabetes. Both roles can foster social integration of adolescents with diabetes into the peer group. The offender role creates social conflicts through discrimination and stigma of adolescents with diabetes. These roles appear during the process of socialization of adolescents with diabetes, where commensality and situations of self-monitoring or administering insulin, key aspect of diabetes treatment, are crucial. Peer groups, depending on the role adopted, may offer support or bring a specific conflict regarding diabetes to their adolescent peer. The combination of roles that friends and peer group play with regards to diabetes will determine the degree of socialization and integration of adolescents with diabetes.

## 1. Introduction

Lifestyle encompasses the whole set of practices undertaken by individuals in their daily life. Among these practices, we may find some which foster a healthier life and others which could be considered risky behaviors [1]. Each individual’s lifestyle will determine the health-illness process, where individual responsibility is essential, and bad habits like tobacco use, poor diet, or physical inactivity contribute to the development of chronic noncommunicable diseases (CNCDs) such as diabetes, high blood pressure, or obesity. From the holistic biopsychosocial perspective, it is crucial to examine lifestyles in a contextualized manner, taking into account the socio-cultural influence in both behaviors and lifestyle sets [2], highlighting the importance, at a clinical level, of the role of those people surrounding us and the context we live in [3].

Having a balanced lifestyle when this disease is diagnosed is crucial to achieve a good diabetes management. Type 1 Diabetes Mellitus (T1D), a paradigmatic case, is a disease caused by a lack of insulin which generates persistent hyperglycemia and energy wasting [4]. At a global level, T1D has a prevalence of 1,110,100 people aged 0–19. In Spain, 15,467 people are affected by this disease [5]. Specifically, in Andalusia its prevalence and incidence have progressively increased, with a total estimated between 30 and 60 thousand people affected [6]. Seville, with a prevalence of 2.06 cases per 1000 population and an incidence of 2396 cases per 100,000 population registered in 2014 is the province with the highest rate [6].

T1D has a multimodal treatment, comprising insulin intensive therapy, diet therapy, physical exercise, self-monitoring, and diabetes education. The complexity of the treatment lies in the continuous requirements that should be incorporated to the individual’s lifestyle. As diagnosis usually takes place at an early age [7] –childhood or adolescence– these individuals are in developmental stages, and therefore still creating their living habits. This is why achieving good glycemic control taking into account the child/adolescent environment from the very beginning is crucial to ensure an optimum quality of life. Failure to adhere to treatment involves a metabolic disorder which may cause serious, chronic complications, risk of high morbidity and mortality, and disabilities [8]. Other chronic diseases, such as obesity, asthma, or epilepsy, may appear in different ranges (mild to severe) [8,9], and though they may require of basal-exposure therapy, it is common for this type of treatment to be occasional and discontinuous [9,10] according to the development of the symptomatology, as it is the case of allergic asthma [10]. In contrast to these chronic diseases, T1D requires of a continuous baseline treatment: constant daily record in which the individual has to make decisions, strict and multiple self-management of blood glucose level, and taking different insulin shots [11]. All this generates emotional stress as a consequence of an active role in self-management [12]. Other diseases which may appear during childhood or adolescence, such as cancer, require of much more aggressive treatments than those related to T1D, though these treatments are applied during long hospital stays [13] and there is a high rate of success in both treatment and cure in developed countries [14,15]. However, in T1D long hospitalizations may be only to the disease debut [16], but treatment requires constant attention within the social and familiar spheres, and nowadays there is no cure [16].

The first years of T1D evolution are a critical stage, significantly more if they overlap with the adolescent stages, when young people struggle to gain autonomy and independence from their families [17,18,19]. In the early stages, adolescents with diabetes are highly motivated to learn about the adequate management of their treatment and comply with adherence [20]. This is driven by their desire of achieving independence from families and gain integration among their peers in the social sphere [21]. However, the adolescent must face the ambivalence between the support and pressure to feel part of the peer group [22], as peer group support, of all socio-cultural influences, has proven to be a key lifestyle aspect at this stage. The adolescent wants to comply with expectations, adopting behaviors which are socially accepted, in order to feel part of the peer group [23], a whole process which leads them to generate a sense of peer group belonging and identity validation [24]. In this sense, adolescents with diabetes may adopt behaviors that diminish previously gained adherence, avoiding treatment requirements in order to obtain a solid integration in the group [25,26,27,28].

## 2. Theoretical Framework and Background

Adolescence is a period of development and evolution for individuals aged 10 to 21 approximately, though nowadays there are no tacit agreements on age range [29]. This stage of constant development can be divided in three age brackets according to physical changes (ages 10–13), acquisition of abstract thinking (ages 14–17), and the quest for self-identity (ages 17–21) [30]. During the whole stage of adolescence there is a reduced perception of risk and a distorted feeling of power [31]. This leads adolescents to believe that they must live in accordance to their feelings instead of accepting advice on the part of health professionals or their own families [32].

Though it may be considered that the strongest support is provided by their families, adolescents may or may not perceive this support, and consider it overprotective [33] or as a lack of understanding on the part of the families [34]. For adolescents, peer groups are very powerful support networks, though they may cause conflict due to the ambivalence between the received support and the social pressure adolescents must face in order to feel part of the group [35]. This is why the current literature points out the importance of peer role and the need of further research in this field [36].

Chronic diseases during adolescence may pose different implications according to the stage affected by the disease. In an early stage, (ages 10–13) adolescents are not concerned about their disease as they are fully focused on adapting to their peers and become part of the group. During an intermediate stage, (ages 14–17) there is a strong concern about self-image [37] and how it is affected by the disease [38,39]. In the late adolescence, (ages 17–21), there is a strong concern about the disease and its complications with regard to social relations and finding a partner [38]. Moreover, a chronic disease in adolescents affect their self-esteem, which is low, while anxiety and depression levels are high [38,40].

In the context of chronic diseases, peer support edges up to backing and emotional support, thus being crucial in the adaptation of adolescents with chronic diseases such as diabetes, to their own disease [41]. This support is essential both for their emotional well-being and their compliance and remains a protective factor in adolescence [42].

In the case of adolescents with diabetes, constant compliance may affect their social relations when acute complications, regular medical consultations, or negative impact in the self-image appear [38]. These situations contrast sharply with their peer’s reality, posing a risk for discrimination. It may be considered that adolescents with diabetes show a potential high risk of not following an adequate self-monitoring of the disease due to two crucial aspects: sense of invulnerability and the competing daily demands of the treatment [43]. For this reason, it is crucial to analyze how peer relations affect group affiliation and self-care of adolescents with diabetes.

As background literature on the relationship between adolescents with diabetes and their peers, there are qualitative studies [22,44,45] with results describing an erratic social support, i.e., sometimes these adolescents receive this support and sometimes they do not. In this respect, the study by Greco et al. [46] offers a joint intervention of an adolescent with diabetes and a friend or peer, showing a considerable improvement in both knowledge and support offered by this peer. However, in spite of the efficacy of the intervention, the author [46] highlights the need of examine adolescents with diabetes from the role of their peers. Some interventions aimed to help adolescents with diabetes to overcome different social barriers have proved effective [47]. Nevertheless, this type of interventions is nowadays limited, as the traditional model of diabetes healthcare, mainly focused on an adequate blood glucose control, prevails. This fact has pushed forward the challenge of acquiring social skills on the part of adolescents with diabetes [48].

Research on how peers influence in the behaviors, both healthy and risky, of adolescents with chronic diseases such as T1D is still not precise enough [24,25,42]. Consequently, the study of peer roles is crucial to survey the group affiliation and self-care of adolescents with diabetes [49].

The objective of this research is to know in depth the social support perceived by adolescents with diabetes from their peers, identifying the roles adopted by peers regarding diabetes, and their influence both on the integration and self-management of the adolescent with diabetes.

## 3. Methodology

### 3.1. Sample and Procedures

A pilot study was based on a descriptive, phenomenological, retrospective, cross-sectional design. With the aim of analyzing in depth the feelings, perceptions and experiences of participants, qualitative methodology with in-depth interviews to key informants was applied. The duration of the interviews was not limited. Interviews were audio recorded and field notes were registered to encompass non-verbal communication aspects.

The study population consisted in adults aged 18–35 living in Andalusia (Spain) with T1D of at least 4 years after diagnosis at the time of the interview. However, as it is a pilot study, the sample was targeted to the province of Seville, the capital of the Andalusian region, benchmark regarding healthcare services, and the province with the highest rate of incidence of T1D in the whole region. Besides, Seville hosts a flagship association of people with diabetes with a youth branch which provided the sample purposely. The field research was accomplished between April and June in 2019.

The first contact among participants and researcher was facilitated by a trusted figure familiar to them: the president of the association youth branch, who acted as key informant. Thus, a snowball sampling was built through this key informant, allowing access to the rest of participants to create a chain-referral, non-probability sampling, so that participants could comply with the sampling inclusion criteria.

Once the interview was agreed with the participant, they were informed of the nature of the study and the informed consent was signed. In-depth interviews were semi-structured, covering the following dimensions: relationship with peer group (number of groups, usual dynamics), Diabetes and social relations with peers (social support perceived), Perception of behaviors of different friends in daily situations (eating, consumption of toxic substances, school/work, leisure moments), and Perception of conflicts with peers regarding diabetes.

Interviews were conducted by the lead researcher, who has a three-year experience as academic intern, plus a year as honorary assistant at the University of Seville, a period in which she worked conducting semi-structured in-depth interviews. This researcher had previous experience in the subject of study after having accomplished different nursing roles with children and adolescents with T1D (Pediatric Nursing consultancy in May 2017, and a Diabetes camp in June 2017).

The sample consisted of 15 individuals with T1D aged 18–35. Data saturation was partially reached with the sixth participant in the dimensions Peer relations and Diabetes and social relations with peers. Data saturation was fully reached with the eleventh interviewee, though four more interviews were conducted in order to bring more consistency and solidity to the study. Given the risk of circumventing sensitive information, intentionally or not, in the presence of a health professional, retrospective research was accomplished working from memory. Though there was the risk of oblivion, the more critical period of adolescence (ages 12–17) was bypassed, resulting in a more holistic vision of the period. In order to bypass the forgetting curve, a maximum age of 35 years old was fixed, as this age marks the consolidation of maturity. Besides, peer relations during adolescence convey highly affective bonds, facilitating their recall [49]. For this reason, the minimum age for participants was set in 18 years old.

T1D diagnosis entails significant changes in both affected individuals and their environment, requiring an adaptation period of even a whole year [50]. Participants have at least a 4-year T1D diagnosis at the time of the interview, thus allowing them to feel fully adapted to their new routines and feel part of the therapeutic regimen. In summary, the selection criteria were: participants aged 18–35, T1D diagnosis during their childhood or early adolescence, and a minimum of 4 years of evolution. Gender influence was not subject to this study; 6 men and 9 women took part in the interviews.

Exclusion criteria were the following: being affected by a psychiatric pathology, being affected by any difficulty which could block the interview communicative process, cognitive impairment or intellectual disability, T2D diagnosis, or gestational diabetes.

### 3.2. Procedure and Ethical Considerations

Each participant was provided with an accessible, voluntary informed consent for them to read and sign, together with an insightful verbal explanation. Interviews were recorded, and therefore they took place in quiet places which guaranteed the comfort and confidentiality of each participant. This study was validated by the Research Ethical Committee of the Hospitals Virgen Macarena-Virgen del Rocío in the session held on 30th April 2019 (CEI 08/2019). In order to guarantee their confidentiality, participants’ quotations appear codified in this manuscript.

### 3.3. Data Analysis

The lead researcher accomplished the interviews transcription, including field notes, and the discourse analysis through those transcriptions. The first step, once the interviews were transcribed, was to extract all the codes which allowed a comprehensively codification of the different discourses according to their similarities. Right after the codified data were grouped according to the phenomenon similarities, the following labels were established: Social Support Perceived and Perceptions.

The category “Social Support Perceived” covers peers’ instrumental or emotional support as perceived by adolescents with diabetes and was analyzed using two variables. The first of these variables, “Peer Role” encompassed behaviors, attitudes, and specific relations with regard to diabetes adopted by the adolescent’s peers. The second one, “Perceptions” comprises the emotions and feelings generated in participants with regard to their peer relations.

Individual differences and the contribution of these peculiarities to the information global significance were taken into account where appropriate. Once each variable was individually analyzed, they were interrelated according to their meanings in order to build a general discourse which corresponded to the study category defined, from which study results could be drawn.

Relaying on the list of references cited [51,52,53], the validity of this qualitative study is achieved in the first place through a critical aim during the exhaustive discourse analysis accomplished by the lead researcher. Secondly, a triangulation regarding the credibility of participants’ perceptions through their discourses was carried out by the different authors. Finally, the authors’ triangulation set the basis to establish the composition of results.

Faced with the impossibility of isolating variables from a complex, holistic reality resulting from a qualitative study [51,52,53], the reliability of the study was accomplished through the triangulation of the recordings together with the field notes (body position, gestures, and speech tone), cross-checked by the different authors. This process substantiated the results found.

## 4. Results

The average age in the sample was 20.33 years, being 8.93 years old the average age of debut of this disease at the time of the diagnosis, with a maximum of 13 years and a minimum of 0 (perinatal period).

Ostensibly, the relationship established by adolescents with diabetes with their peers is similar to the one established by any other adolescent with his or her group of friends. This implies that they can benefit from different types of support (emotional, instrumental, and informative) offered by the social network shaped by the peer group, and therefore improving the adolescent well-being while helping to cope with stressful situations. But when focusing on specific support regarding this chronic disease, we may observe how certain roles emerge in the attitudes of the peers resulting from diabetes management: capillary blood glucose testing, carbohydrate counting, insulin injections, and complication management in case of hypoglycemia, for instance. When a group member has T1D, peers adopt specific behaviors in different scenarios. Depending on how these attitudes are perceived by adolescents with diabetes, three types of specific peer roles can be distinguished in relation to diabetes: protective role, indifferent role, offensive role. This section may be divided by subheadings. It should provide a concise and precise description of the experimental results, their interpretation, as well as the experimental conclusions that can be drawn.

### 4.1. Protective Role

The protective role embodies a set of peer attitudes and behaviors which foster self-management, favoring peer affiliation. In general, a protective role fosters behaviors which promote healthy habits in adolescents with diabetes, favoring self-control and fostering appropriate behaviors towards therapeutic compliance.

Sometimes when they saw me... I don’t know, eating ice-cream, they’ve asked: ‘Hey, Are you allowed to eat that?’ Or they’ve said: ‘Don’t you need an injection?’ or ‘Are you watching your dose?’ Then yes, maybe I didn’t get it right, so they were trying to help to control it even better.

* (Interview -I- 8)*

With regard to nutrition, peers try to help adolescents with diabetes to avoid unhealthy food or those which are not recommended, without breaking the group dynamics, that is, peers also consume these types of food. Though food dynamics continue in the group, supportive attitudes arose among these peers: intake restrictions, previous warnings, food planning specifically devised for the adolescent with diabetes, etc.:

As they are aware of my disease, they don’t prepare high-carb food, and use much more greens. For example, the other day I was having lunch at a friend’s and they prepared me a minced salad dish with tomato, onion, green pepper, vinegar and tuna... I ate it all. They ordered some pizzas...he he.

*(I14)*

A delicate situation for adolescents with diabetes is related to alcohol consumption. In general, affiliation is fostered through responsible drinking either lead by adolescents with diabetes themselves or their closest circle. Though these attitudes may be questioned as protector roles because they encourage alcohol consumption, adolescents with diabetes identify them as supportive. Two people interviewed acknowledge that, though their friends tried to prevent them from drinking alcohol, they rarely succeeded.

‘Test yourself, yo.’ ‘Hey, eat something so you don’t get sick.’ ‘Girl, check your sugar...’ But it was not like ‘Don’t drink at all’ but ‘If you do it, do it right.’ And that’s support; my friends are supportive with this issue.

*(I11)*

I always said: ‘I can’t drink’ But then I always did it, so they say: ‘Next week no alcohol because it’s bad for her.’ But then the weekend came and they drink again, and I did the same.

*(I13)*

Peers adopting the protective role during these moments try to suggest the type of alcoholic and non-alcoholic beverage (i.e. light soft-drinks or Coke Zero^®^) in order to adapt consumption to the requirements of their friend with diabetes.

Well, my friends care, right? And if they have to buy Coca-Cola they bring Zero^®^, but when... a Mojito bottle is five euros and gin is seven, and if you mix mojito and 7UP we had a bowl like this [arms wide open] and gin is just for two... I cannot impose to buy what I want.

*(I11)*

In the context of self-care, adolescents with diabetes need to perform capillary blood glucose testings, take insulin injections or use an insulin pump, all of them procedures that often have certain impact on both self-esteem and self-image. It is common for adolescents with diabetes to hide these practices their peers, out of shame or embarrassment, but there is usually a close friend to share these situations with, who plays a protective role offering company and emotional support.

I was ashamed of giving myself a shot in front of them, I don’t know. When I needed my insulin dose, I used to wait until one of my girlfriends needed to go to the bathroom […] I normally used to go with a friend who knew... A close friend.

*(I10)*

Besides the closest friend, the rest of peers also remind adolescents about the need of monitoring insulin administration, and they even offer themselves to perform the procedure thus learning about treatment management. Though adolescents with diabetes prefer to undertake themselves their self-management, this type of attitudes on the part of the peer is really appreciated. Peers who adopt a protective role get genuinely involved and actively ask for information about how to proceed.

My friend asks me to show her everything, you know, testing, insulin, Glucagon, whether if she needs to call an ambulance...

*(I7)*

The fear of hypoglycemia is perceived in most of discourses, and specifically peers show concern about glycemic control and about how their diabetic friend feels. As they become familiar with the situation, they are able to anticipate hypoglycemia episodes, warning their friend or alerting people around, or they act directly providing sugary foods.

All my groups of friends have been informed about this, so they have been helpful. So, when they feel I’m acting weird they always realize and tell me that my sugar is low.

*(I3)*

Also, if I felt dizzy when I was with my classmates, they told our teachers about it […] They even explained them everything, I didn’t have to tell my teacher that I needed to eat something.

*(I9)*

### 4.2. Indifferent Role

The indifferent role involves lack of actions or supportive behavior with respect to diabetes, therefore letting adolescents to be autonomous and self-caring. In contrast to the protective role, peers adopting indifferent roles do not try to avoid certain behaviors on the part of the adolescent with diabetes, which could translate into lack of health-promoting attitudes. However, this is perceived by adolescents with diabetes as inclusive, as no difference with respect to the peer group is highlighted. Moreover, adolescents with diabetes perceive indifferent roles as an opportunity of feeling independent, in contrast with attitudes perceived from their families.

If they have to help me they would, but I was my business, they cannot be like ‘Did you take your insulin?’ [silence] No. […] But it is something they were clear about, and they knew I had to take full responsibility. It was mine and no one else’s.

*(I2)*

Though indifferent roles imply the absence of direct actions, adolescents with diabetes describe these peers as informed and aware of procedures in the case of complications, while respectful of their autonomy:

Normally they don’t say anything about it, but I knew they were... watchful, you know? They were observing me, watching this or that, but saying nothing... Well, except if something happened, and then they did something. But in general, I could be on my own, and they were ok.

*(I6)*

### 4.3. Offensive Role

Offensive roles adopt a discriminatory behavior towards adolescents with diabetes merely because they suffer this disease and need to undertake self-care. Consumption of food identity markers such as hamburgers and pizzas represents a source of conflict for these adolescents. However, food with high levels of simple carbohydrates becomes the major source of discrimination, thus perpetuating all diabetes myths and beliefs about not consuming any sugar. Some peers adopting the offensive role are self-assured about the impossibility of sugar consumption on the part of adolescents with diabetes. They take for granted the fact that these adolescents are not going to consume this type of food, and therefore they do not have to share it. These situations are usually managed in a humorous context, but adolescents with diabetes may perceive them as a mockery.

Then they started with cruel jokes associated to diabetes, typical of people at that age, right? [...] If we decided to go to a sweet shop, and maybe I did not want to buy anything, but just hang with them, as we did every Friday. But then one of them told me that I could not go because I was diabetic. And I was thinking like ‘is he making fun of me or is he trying to protect me? And I said him: ‘Don’t you worry, I’m not buying candy.’ And he was like: ‘No, I say you are not allowed in because you are diabetic.’ And then he said: ‘Look, the diabetic wants to enter there...’ [mocking tone] I was crossed with him, like forever.

*(I1)*

Besides nutrition, other situations that lead to discrimination of adolescents with diabetes are those related to insulin therapy and glycemic control. Many times, peers adopting the offensive role deliberately compare adolescents with diabetes to drug users, due to injection practice. This connection is established both inside and outside the school context and usually takes the form of scorn and derision, displaying a whole range of offensive terms to address them.

When I’m in the street and I need a shot, they shout to me: ‘Don’t smash yourself here, you crackhead’, and that kind of stuff, I felt embarrassed. But also [clears her throat] at the beginning, I was afraid. I was afraid of being different, you know? What really worried me, mmmh... was that people gave me weird looks when I needed my insulin injection, or that they stared at me.

*(I11)*

In this situation, the adolescent with diabetes claims to have felt bewildered, as she cannot fathom that kind of comparison with regards to a therapeutic procedure (insulin injection) essential for her health. Frequently, these experiences may be potentially painful for adolescents with diabetes, who confess feeling scared and embarrassed. Besides, these situations may lead to a conflict, direct or indirect, or to avoid social contact with peers adopting these attitudes. Thus, it can be concluded that offensive roles adopted y peers do not favor group affiliation and do not offer social support, on the contrary, it may lead to situations of verbal or physical abuse.

Such is the emotional impact of the offensive role on adolescents with diabetes that it may endanger their health due to self-care negligence. A common illustration of this occurs when peers ridicule hypoglycemia symptoms and manifestations, causing adolescents to feel embarrassed and therefore avoiding any action to reverse this situation, in order to avoid subsequent mockery. Definitively, offensive role favors situations of exclusion and undermines psychosocial well-being of the adolescent with diabetes.

I have had to fake how I was feeling when my sugar was dipping low. So they don’t say … [sad tone] so they don’t laugh at me, as some of them did when I was all sweaty and white, because this is how you look, pale as a sheet.

*(I12)*

## 5. Discussion

In their process towards social integration, adolescents with diabetes aim to follow group dynamics trying not to feel different with respect to their peers [54,55]. Up to this point, previous researches [56,57] try to relate peer influence to treatment adherence, being this relation still limited [58]. In this sense, peer support does not appear clearly linked to an optimal glycemic control, but peer conflict is closely linked to a worsening of glycemic control and self-care [58]. Thus, conflict may be considered to cause a deeper impact than support [59]. De Wit et al. [60] systematic review claims that family support to adolescents with diabetes is nowadays a well-defined contribution. However, it is not clear if peer influence is negative or positive as, on the one hand, peers may complement the support offered by the families, but also, social conflict among peers (incongruity between the behaviors of the adolescent with diabetes and those in the group) may lead to negative results regarding diabetes.

Further evidence on how peers influence health and risk behaviors in adolescents with CNCDs [25,56] is still needed. In this sense, La Greca et al. [25], highlight the need of an inside appraisal on how and why adolescents with a CNCD succeed or fail in their social relations, and here the study of role is crucial.

With respect to these roles, Rankin et al. [61] and Kyngäs et al. [62] highlight the presence of similar role to the ones observed in this study (Table 1). In the study by Rankin et al. [61] peer roles are classified into three types of support: normalizers, monitors and prompters, and helpers. Those who do not offer support are labeled as insensitive and unsupportive peers. For their part, Kyngäs et al. [62] distinguish three supporting roles in their study: dominating, silent, and irrelevant.

Silent support, described by Kyngäs et al. [62], consists in a group dynamic change towards a healthy lifestyle, avoiding some types of food such like sweets, normalizing diabetes and facilitating peer integration. This study has failed to observe significant changes in group dynamics regarding the protective role, but in food-related contexts they tried to reduce the intake of adolescents with diabetes, or even try to prepare something more appropriate for healthy eating. In second instance, the roles of normalizers, monitors and prompters, and helpers proposed by Rankin et al. [61] come into play in self-management situations (capillary blood glucose testing and insulin administration), complications (basically, potential or real hypoglycemia), and in general as emotional support and backing. Similarly, qualitative studies by Comisariado et al. [22] and Marshall et al. [44] highlight that, in some cases, when adolescents reveal their diabetes diagnosis, they receive social support on the part of their friends and this foster positive attitudes on the part of the peers. All these support actions are also encompassed by the protective role and foster group integration.

This definition of indifferent role differs from the irrelevant support proposed by Kyngäs et al. [62], which contemplates no direct peer influence according to the perceptions of adolescents with diabetes. On this regard, this research cannot deny the possibility of this influence because although it is not implicitly manifested in any discourse, certain degree of gratitude towards the peer group is observable, and therefore some influence may be deducted. Pendley et al. [45] observed that peers’ lack of specific knowledge about daily management of diabetes, what may result in two types of behaviors: absence of support or neutral support. Neutral support, coincident with the indifferent role presented here, would consist on not establishing a differentiating barrier between the adolescent with diabetes and the peer group, therefore fostering inclusion, but with bringing a duality: the adolescent with diabetes may perceive this behavior as a form of emotional support which encourages risk behaviors or behaviors which do not favor self-care. In this sense, Marshall et al. [44] highlight that, according to the perception of adolescents with diabetes, the support perceived is limited due to their peers’ lack of training.

I don’t know, I never felt they were doing a fuss around my issue, I mean, they maybe asked me ‘How are you?’ ‘Can you eat this?’. Those are things I’ve been asked... But just once at a time, when I answered them, that was the end of it.

*(I15)*

The insensitive role proposed by Rankin et al. [61] is similar to the offensive role in this study: peers show no empathy and discrimination appears. In contrast to last analysis [61], this research fund evidence of humiliating attitudes and insults, resulting in self-care restraint in order to avoid peer rejection. This situation is also described by Gürkam et al. [63] showing that distress, frustration, and helplessness lead adolescents to hide their diagnoses.

When meeting someone new, I never say: ‘Hello, I’m diabetic’, you know what I mean? […] I’m not comfortable at all saying I have diabetes.

*(I11)*

Finally, the dominant role described by Kÿngas et al. [62] involves peer pressure so adolescents with diabetes feel ‘forced’ to follow group behaviors ignoring their disease in order to comply with social integration or treatment adherence. Although there is no direct correlation with the roles proposed in this study, the dominant role [62] is to some extent present in the behaviors adopted by both protective and indifferent peer roles here described. On the one hand, peers encourage the adolescent with diabetes to carry out group practices, including the consumption of toxic substances, -in a controlled way- under the protective role. On the other, if peers adopt an indifferent role and do not get involved, self-caring on the part of the adolescent may be neglected in favor of group dynamics. Ultimately, offensive roles may cause adolescents compliance with peer group by means of a passive behavior, for fear of reprisals or rejection. Under this assumption, this may cause a dominant behavior such as the role suggested by Kyngäs et al. [62].

Most studies on peer influence in children or adolescents with diabetes do not cover the use of alcohol and other drugs, as they focus on the treatment requirements (exercise, diet, insulin therapy, and capillary blood glucose testing), glycemic control, and adherence and quality of life achievements [56,59,61,62].

Finally, with respect to the offensive role and discrimination, there is a lack of scientific evidence [56,59,62]. The insensitive role observed by Rankin et al. [61], shows similar behaviors, although analyzing an underage sample (preadolescents) results are slightly different.

According with the results, adolescents with diabetes ascribe great importance to the fact of feeling ‘normal’ and not different from their peers and, unfortunately, they show a limited social success [22]. Continuous self-caring and hypoglycemia disruptions easily cause stigma, becoming bullying targets and conflict situations all practices associated to disease management (capillary blood glucose tests, insulin injections and dietary restrictions) [58].

One of the stigmas more commonly highlighted by adolescents with T1D is the lack of information around the disease, which consequently it is linked to the impossibility of consuming certain foods or performing certain actions [64]. According to Browne et al. [65] adolescents with diabetes suffer from a lack of knowledge and misinformation of the rest of the population, which may be caused by wrong media outreach. Consequently, during friend meals, adolescents with diabetes have to cope with social prohibitions, resulting in a negative impact on their identity caused by their peer’s failure to differentiate the recommended guidelines for the disease. Therefore, inner conflict arouses and some adolescents avoid disclosing their disease [22,65,66] as revealed by participants.

People sometimes are a pain in the neck [...] It’s a shame that due to misinformation, uhm... they are bothering you and in the end making you feel really bad. ‘Why are you eating that?’ And then you have to explain everything, all the time… It’s like, I eat this because I want, damm. ‘But you can’t.’ Well, I can, and that’s the end of it. It’s exhausting.

Ultimately, the results obtained allow an overview of both social support as perceived by adolescents with diabetes and the identification of peer roles. The influence of these roles, rather vague in previous literature [22,56,59,60,62] has been clarified in this pilot study. In contrast to results obtained in other studies [44,45,62], the present wok observes that the protector role not only foster healthy and self-care behaviors which facilitate integration, but also it may develop a crucial role in common scenarios of adolescence (toxic substances consumption, for example) encouraging a ‘controlled’ consumption to facilitate and improve the integration of the adolescent with diabetes. However, though this attitude may favor integration, it has a negative impact on the adolescent’s self-care. This duality is also observable in the indifferent role, matching the results of the study by Pendley et al. [45]. By contrast, this role duality has not proved to be so far the origin of the discussion on whether or not social support from peers is a positive influence for adolescents with diabetes.

### Limitations

The limitations of this study should be acknowledged. Firstly, that the research is a pilot study, and it has only realized in Seville. Despite we have reached the saturation point, our sample is geographically limited. Secondly, we chose our sample on the basis of convenience, which makes it difficult to extrapolate wider conclusions from the results obtained. Thirdly, this study has focused on the perception of adolescents with diabetes, and no peer interviews were conducted. An analysis of peers’ perceptions would provide a contrasting and therefore in order to provide further conclusions to the study of peer affiliation in the case of adolescents with diabetes. Finally, during some interview, participants acknowledged expectations about what the ideal behavior of their peers could be. However, this aspect requires of another in-depth study given the myriad possibilities conveyed by participants’ subjectivity.

## 6. Conclusions

Peer influence through specific roles is crucial for the group affiliation of adolescents with diabetes. On the one hand, both the protective and indifferent roles facilitate the integration of the adolescent with diabetes. The protective role also fosters controlled consumption of food identity markers and/or alcohol. The indifferent role ignores the disease, without realizing the personal consequences of these practices. Though seen as supportive on the part of adolescents with diabetes, these peers pose a dilemma for adolescents, who have to choose between following common social practices in order to feel part of the group or lead a healthy lifestyle. On the other hand, the offensive role generates stigma and social conflict, which it is not conducive to the integration of adolescents with diabetes, even jeopardizing their physical and emotional well-being. This type of offensive behaviors, according to adolescents with diabetes, may be the result of society’s lack of health information and education regarding T1D.

The number of studies evaluating the how adolescents with diabetes perceive their peers’ behaviors is very limited, and in no case this literature shows a result categorization as simple and comprehensive as the one in this pilot study, which highlights the importance of the duality conveyed by both indifferent and protector roles in the behavior of adolescents with diabetes. Thus, the study offers a possible explanation on why qualitative studies have failed so far in completing the overview on peers’ influence in terms of positive or negative influence.

The innovative approach focuses on the results regarding the protector and indifferent roles and the possibility that they may introduce, simultaneously, both a positive and negative influence. This fact enables our study as a foundation for further, more extensive research which may not only confirm these roles duality, but also provide an in-depth, extended analysis of the offensive role and its consequences.

The practical implications of this research may be observed at several levels. In the field of research, it offers a possible explanation to a phenomenon hitherto unclear. Expressly, the acknowledgment of specific peer roles facilitates precise health care interventions which will help to improve not only the affiliation of adolescents with diabetes, but their coping with social conflict scenarios, therefore improving their psychosocial well-being.

In the field of education, it is possible to offer a conceptual benchmarking framework on the type of behaviors that may be generated in the class context towards the student with diabetes. This could facilitate the planning of prevention strategies on the part of teachers in order to avoid discriminatory attitudes posed, for example, by the offensive role. Therefore, these professionals could foster the integration of adolescents with diabetes in the class. Together with teacher training, the applications of this study could facilitate understanding of parents and families of adolescents with diabetes. Adolescence is a critical time, where knowing what the adolescent is really doing or how they cope is very difficult for parents, since adolescents rely on the group of friends for confidence and comfort. Therefore, observing the possible behaviors that these friends may adopt will help parents to guide them, understanding some behaviors and the outcomes regarding diabetes at this stage. With respect to adolescents with diabetes, knowing in advance the roles of peers within their social sphere provides them with the possibility of working in advance on coping strategies for the different role behaviors.

When approaching this knowledge from a multidisciplinary perspective (educational sciences, health, and psychology) together with their family, adolescents with diabetes may achieve a considerably more effective group integration.

Finally, it is important to be realistic when appraising the adoption of self-care behaviors in adolescents with chronic diseases. Even in the best scenario cases of social integration, and when peers are well informed about the disease, adolescents with diabetes can easily adopt risky behaviors perceived as “controlled” by them and their peers.

## Figures and Tables

**Table 1:** Peer behaviors.

Roles Defined in our Research	Type of Support	Friends Actions
	Proposed by	Proposed by
	Rankin et al. [61]	Kyngäs et al. [62]
Protective Role	Monitor Prompters	Silent Support
	and Helpers	
Indifferent Role	Normalizers	Without Significance/
		Irrelevant
		(Good Compliance)
Offensive Role	Insensitive and	Domination
	Unsupportive	(Poor Compliance)

## Data Availability

The data are not publicly available due to privacy or ethical considerations.
